# Diagnostic accuracy of BRCA1–associated protein 1 in malignant mesothelioma: a meta-analysis

**DOI:** 10.18632/oncotarget.20317

**Published:** 2017-08-17

**Authors:** Li-Ming Wang, Zhen-Wang Shi, Ji-Ling Wang, Zhi Lv, Fang-Bin Du, Qing-Bin Yang, Yong Wang

**Affiliations:** ^1^ Department of Gastroenterology, Second People's Hospital of Hefei, Anhui, China; ^2^ Department of Respiratory Medicine, Second People's Hospital of Hefei, Anhui, China

**Keywords:** diagnostic accuracy, diagnostic accuracy, malignant mesothelioma, malignant mesothelioma, BAP1

## Abstract

**Background:**

Conventional measurements are not always helpful in the diagnosis of malignant mesothelioma (MM). Increasing studies indicate that loss of BRCA1–associated protein 1 (BAP1) detected by immunohistochemistry (IHC) is a useful diagnostic marker for MM. In this meta-analysis, we investigated the diagnostic accuracy of BAP1 in MM.

**Results:**

In total, 12 eligible studies with a total of 1824 patients were selected. Results indicated that loss of BAP1 sustained a pooled sensitivity of 0.56 (95% CI, 0.50–0.62), specificity of 1.00 (95% CI, 0.95–1.00), PLR of 548.82 (95% CI, 11.31–2.7 × 104), NLR of 0.44 (95% CI, 0.39–0.50), DOR of 1247.78 (95% CI, 25.08 −6.2 × 104) in discriminating MM from non-MM. The AUC of 0.72, reflecting the SROC, indicated moderate diagnostic accuracy. Subgroup analysis showed that BAP1 detection in histological specimens owned the higher diagnostic performance than cytological ones. In addition, BAP1 showed superior diagnostic accuracy in epithelioid MM than biphasic or sarcomatoid MM.

**Materials and Methods:**

PubMed, Embase and the Cochrane Library and reference lists of related articles were searched, and studies that evaluated the utility of BAP1 in MM were included. Data from eligible studies were pooled to estimate sensitivity, specificity, positive likelihood ratio (PLR), negative likelihood ratio (NLR), diagnostic odds ratio (DOR). Summary receiver operating curves (SROC) was applied to estimate overall diagnostic accuracy.

**Conclusions:**

Current meta-analysis indicates that detection of BAP1 by IHC is a useful diagnostic marker for MM. Loss of BAP1 almost provides confirming diagnosis for MM, while positive staining for BAP1 is not enough to exclude non-MM.

## INTRODUCTION

Malignant mesothelioma (MM), primarily linked to asbestos exposure, is a highly aggressive cancer that metastasizes easily, and MM patients usually have poor prognoses [1, 2]. MM originates from the mesothelial lining cells of serosal membranes, including the pleural, peritoneum, pericardium, and tunica albuginea [3]. MM is more common in the pleura of elderly men in the western world, especially in Australia and Europe, in which patients suffering from this typically present with unexplained pleural effusion and chest pain, accompanied by weight loss and fatigue [4]. Although different therapeutic options for MM exist, there are few available effective options and its prognosis remains poor [5–7]. The likelihood of survival one year beyond diagnosis is < 50 percent; thus, it is critical to accurately detect MM in early stages [1]. However, the diagnosis of MM is still difficult, and the challenging problem is distinguishing MM from benign mesothelial proliferation or adenocarcinoma on slides stained with haematoxylin and eosin. Imunohistochemistry (IHC) can provide a definitive MM diagnosis [8]. Several markers (such as p16) have been recommended in order to support the MM diagnosis [9]. However, very specific and sensitive markers for mesothelioma are absent [10].

The *BRCA1–associated protein 1* (*BAP1*) gene, located on chromosome 3p21.1, is a tumour suppressor gene, and germline BAP1 mutations have been found in association with hereditary cancer syndrome. Loss of *BAP1* is frequently observed in MM and has been proven to increase the risk of MM [11–14]. BAP1 protein is a deubiquitinating enzyme, which serves as a tumour suppressor via regulation of DNA damage repair, cell cycle, and cellular differentiation. In recent years, the loss of the BAP1 protein, detected by IHC, has been widely studied for diagnosing MM. However, the results are varying, and the role of BAP1 detection in MM is still controversial. To establish whether BAP1 detection could serve as a useful tool in the diagnosis of MM, we performed a systematic review and meta-analysis by pooling relevant published studies. In addition, we assessed the diagnostic performance of BAP1 and MM subtypes on cytological or histological specimens.

## RESULTS

### Study characteristics

**Figure 1:**
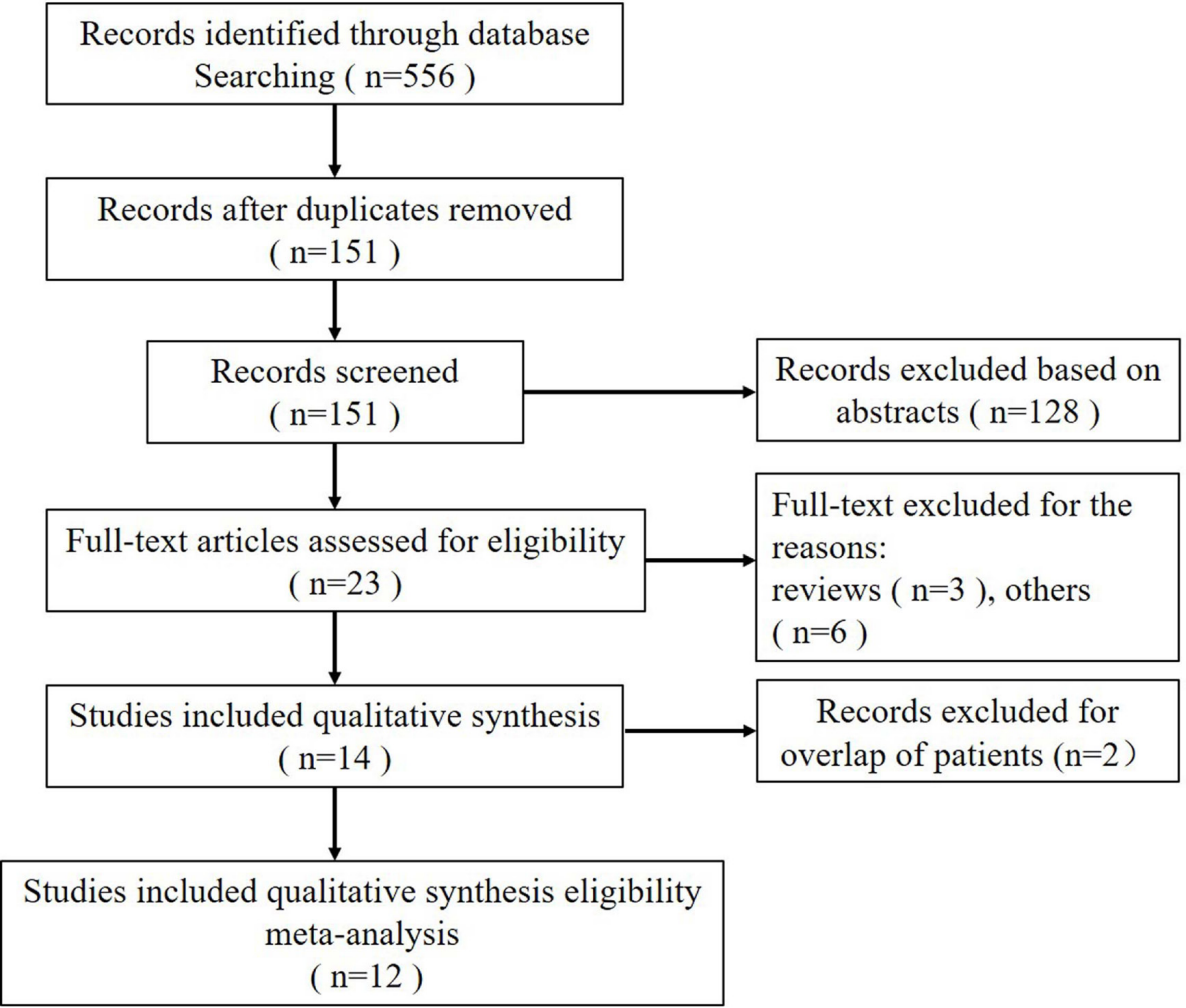
Flow diagram showing inclusion and exclusion of studies

After considering independent reviews, 12 studies were included in the meta-analysis [15–26]. A flow chart, which included the reasons for study exclusion, is shown in Figure 1. Five-hundred fifty-six publications, of which 405 were duplicate studies, were obtained from the database search. After screening titles and abstracts, 128 studies were excluded for their contents that were not relevant to our study leaving 23 eligible articles. Of these, three publications were excluded because they were reviews, and six were excluded because the sensitivity or specificity calculations could not be allowed. Also, since two studies [27, 28] reported patient overlap with two other studies [22, 24], we chose studies containing larger subject samples or with the best quality to avoid the duplication.

Finally, 12 eligible studies with a total of 1824 patients were enrolled for the analysis. The characteristics of the eligible studies are presented in Table 1. The selected studies were published between 2015 and 2017 and included 1016 patients with confirmed MM and 808 patients with non-MM. The non-MM included non-small cell lung cancer, reactive mesothelial hyperplasia, benign mesothelioma, fibrous pleurisy, and others. All 12 studies were retrospective in design. Briefly, the types of sample are histological or/and cytological. There were two studies [16, 23] of which cytological and histological data reported an MM overlap. Although the data between the two types were almost the same in two studies, we chose histologial data for the reason that cytology cannot always provide a definitive diagnosis [1]. Most publications did not set cut off values for the loss of BAP1 staining while the loss was defined as nuclear staining of < 19.4% mesothelial cells in the study by Hida [22]; Shinozaki-Ushiku [25] set nuclear staining as < 10.0% mesothelial cells, and the study by Walts [26] defined absence of nuclear staining in > 50% atypical cells.

**Table 1:** Characteristics of the included studies

Study	Study type	Sample Type	Diagnosis/Site	Sample size MM/Non-MM	Antibody
Andrici 2015	Retrospective	Cytology	Pleural	75/47	Clone C-4, Santa Cruz
Cigognetti 2015	Retrospective	Histology Cytology	Pleural and peritoneum	218/48 45/17	Clone C-4, Santa Cruz
Sheffield 2015	Retrospective	Histology	Pleural, peritoneum and pericardial membrane	31/52	Clone C-4, Santa Cruz
McGregor 2015	Retrospective	Histology	Pleural	111/20	Clone C-4, Santa Cruz
Carbone 2016	Retrospective	Histology	Pleural	35/45	Clone C-4, Santa Cruz
Walts 2016	Retrospective	Cytology	Pleural or peritoneum	32/35	Clone C-4, Santa Cruz
Andrici 2016	Retrospective	Histology	Pleural or peritoneum	286/395	Clone C-4, Santa Cruz
Hwang 2016	Retrospective	Histology Cytology	Pleural or peritoneum	15/3 15/3	Clone C-4, Santa Cruz
Jaouen 2016	Retrospective	Histology Cytology	Pleural	25/21 23/18	Clone C-4, Santa Cruz
Shinozaki-Ushiku 2017	Retrospective	Histology Cytology	Pleural or peritoneum	32/44*	Clone C-4, Santa Cruz
Guo 2017	Retrospective	Histology	Pleural or peritoneum or tunica vaginalis	22/35	Clone C-4, Santa Cruz
Hida 2017	Retrospective	Histology	Pleural	51/25	Unclear, Santa Cruz

*Including histology and cytology.

### Quality assessment

The assessment by Quality assessment of diagnostic accuracy studies-2 (QUADAS-2) was summarized in Table 2. Twelve and four studies were judged to show an unclear risk of patient selection and index test, respectively, and three studies generated a high risk of bias in flow and timing. The QUADAS in the other studies displayed high quality.

**Table 2:** Details of quality assessment by the QUADAS-2

		Risk of bias			Applicability concerns		
Study	Patient selection	Index test	Reference standard	Flow and timing	Patient selection	Index test	Reference standard
Sheffield 2015	U	L	L	L	L	L	L
McGregor 2015	U	L	L	L	L	L	L
Cigognetti 2015	U	L	L	H	L	L	L
Andrici 2015	U	L	L	H	L	L	L
Hwang 2016	U	U	L	L	L	L	L
Carbone 2016	U	U	L	L	L	L	L
Jauoen 2016	U	L	L	L	L	L	L
Andrici 2016	U	L	L	H	L	L	L
Walts 2016	U	L	L	L	L	L	L
Guo 2017	U	L	L	L	L	L	L
Shinozaki-Ushiku 2017	U	U	L	L	L	L	L
Hida 2017	U	U	L	L	L	L	L

L, low risk; U, unknown risk; H, high risk.

### Diagnostic accuracy

**Figure 2:**
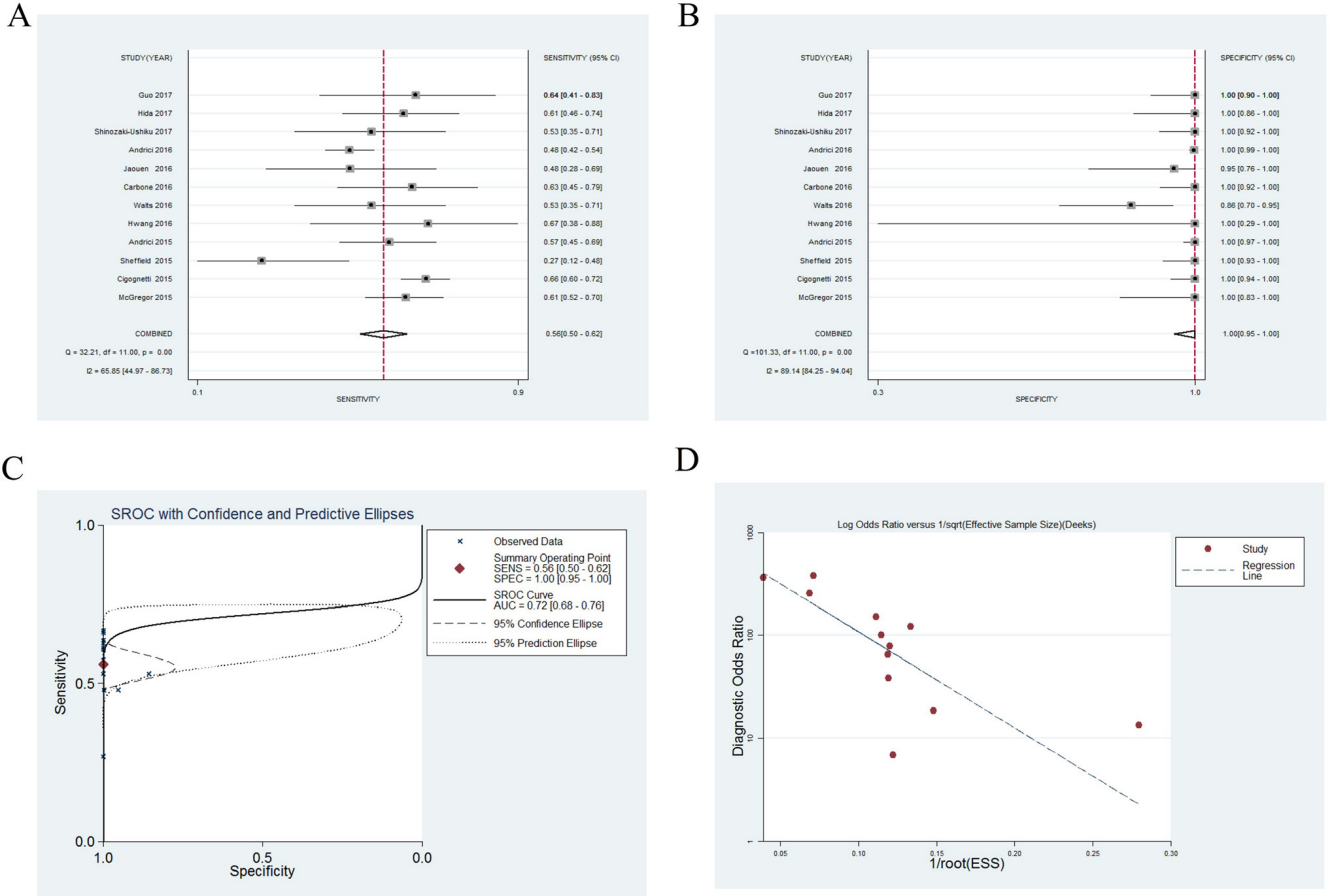
Forest plots for diagnostic accuracy of BRCA1–associated protein 1 (BAP1) on the diagnosis of malignant mesothelioma Sensitivity (**A**), specificity (**B**), summary receiver operative curves (**C**), funnel plot (**D**).

Figure 2 showed that the sensitivity and specificity of BAP1 staining in MM diagnosis were 0.56 (95% CI, 0.50–0.62) and 1.00 (95% CI, 0.95–1.00), respectively. The specificity in 10 studies was 1.00, and the sensitivity in most studies was around 0.57. Only one study showed relatively low sensitivity (0.27). We also noted that pooled positive likelihood ratio (PLR) was 548.82 (95% CI, 11.31–2.7 × 10^4^), pooled negative likelihood ratio (NLR) was 0.44 (95% CI, 0.39–0.50), and pooled diagnostic OR (DOR) was 1247.78 (95% CI, 25.08–6.2 × 10^4^), while the *Q* test *p* value = 0.00 and *I*^2^ = 87.00% (95% CI, 73.38–100.00) showed a high heterogeneity for diagnostic accuracy (Table 3).

**Table 3:** Comparison of diagnostic accuracy of BAP1 detected in cytological specimens and histological specimens

	Cytological specimens	Histological specimens
Number of Studies	5	10
Sensitivity (95% CI)	0.58 (0.50–0.65)	0.55 (0.49–0.61)
Specificity (95% CI)	0.96 (0.89–0.99)	1.00 (0.98–1.00)
PLR (95% CI)	20.53 (8.73–62.17)	180.91 (35.48–6245.84)
NLR (95% CI)	0.45 (0.37–0.54)	0.45 (0.40–0.51)
DOR (95% CI)	53.47 (18.42–170.91)	423.81 (63.48 −1.4 × 10^4^)
AUC (95% CI)	0.69 (0.66–0.73)	0.75 (0.73–0.80)
Heterogeneity	*Q* test, *p* = 0.30;*I*^2^ statistic = 0.00	*Q* test, *p* = 0.00; *I*^2^statistic = 80.11

AUC, area under curve; DOR, diagnostic OR; NLR, negative likelihood ratio; PLR, positive likelihood ratio.

In the summary receiver operating characteristic (SROC) graph, each point represents sensitivity against specificity in a single study, exhibiting test performance summary and trade-off between sensitivity and specificity [29]. As shown in Figure 2, the area under curve (AUC) value was 0.72 (95% CI, 0.68–0.76), indicating moderate diagnostic accuracy.

### Analysis of publication bias

A funnel plot and Egger's test were applied to assess publication bias. The funnel plot illustrated that the point distribution was asymmetric (Figure 2), which was further confirmed by the Egger's test (*p* = 0.001). These results showed that publication bias existed.

### Subgroup analysis

Ten studies [15–18, 20–25] and five studies [16, 19, 20, 23, 26] provided data for evaluating the diagnostic accuracy of BAP1 in histological and cytological specimens, respectively. For the cytological specimens, there were no significant differences in the sensitivity or specificity compared with histological specimens (Table 3). However, the values of the DOR and AUC showed BAP1 detection in histological specimens demonstrated better discriminating capability than cytological ones.

Malignant mesothelioma is classified into three subtypes: 1.) epithelioid malignant mesothelioma (EMM); 2.) biphasic malignant mesothelioma (BMM); and 3.) sarcomatoid malignant mesothelioma (SMM). We also calculated the diagnostic accuracy of BAP1 detection in these three subtypes (Table 4). Five studies [15, 20, 21, 24, 25] provided the data for the three subtypes. For EMM, the values of sensitivity, specificity, DOR, and AUC were 0.74 (95% CI, 0.66–0.80), 1.00 (95% CI, 0.53–1.00), 2293.52 (95% CI, 2.93–1.8 × 10^6^), and 0.79 (95% CI, 0.75–0.82), respectively. For BMM, the values of sensitivity, specificity, DOR, and AUC were 0.50 (95% CI, 0.38–0.62), 1.00 (95% CI, 0.45–1.00), 726.23 (95% CI, 0.83–6.4 × 10^5^), and 0.58 (95% CI, 0.54–0.62), respectively. For SMM, the values of sensitivity, specificity, DOR, and AUC were 0.07 (95% CI, 0.00–0.72), 1.00 (95% CI, 0.49–1.00), 52.52 (95% CI, 0.03–8.4 × 10^4^), and 0.86 (95% CI, 0.83–0.89), respectively.

**Table 4:** Comparison of diagnostic accuracy of BAP1 in the epithelioid malignant mesothelioma (EMM), biphasic malignant mesothelioma (BMM), and sarcomatoid malignant mesothelioma (SMM)

	EMM	BMM	SMM
Number of Studies	5	5	5
Sensitivity (95% CI)	0.74 (0.66–0.80)	0.50 (0.38–0.62)	0.07 (0.00–0.72)
Specificity (95% CI)	1.00 (0.53–1.00)	1.00 (0.45–1.00)	1.00 (0.49–1.00)
PLR (95% CI)	608.72 (0.83–4.5 × 10^5^)	364.95 (0.41–3.2 × 10^5^)	49.00 (0.03–7.0 × 10^4^)
NLR (95% CI)	0.27 (0.21–0.34)	0.50 (0.40–0.64)	0.933 (0.733–1.19)
DOR (95% CI)	2293.52 (2.93–1.8 × 10^6^)	726.23 (0.83–6.4 × 10^5^)	52.52 (0.03–8.4 × 10^4^)
AUC (95% CI)	0.79 (0.75–0.82)	0.58 (0.54–0.62)	0.86 (0.83–0.89)
Heterogeneity	*Q* test, *p* = 0.01; *I*^2^ = 75.87	*Q* test, *p* = 0.01; *I*^2^ = 76.47	*Q* test, *p* = 0.10; *I*^2^ = 36.66

AUC, area under curve; DOR, diagnostic OR; NLR, negative likelihood ratio; PLR, positive likelihood ratio; EMM, epithelioid malignant mesothelioma; BMM, biphasic malignant mesothelioma; SMM, sarcomatoid malignant mesothelioma.

## DISCUSSION

In this systematic review and meta-analysis, we achieved a comprehensive summary of the recently published studies investigating BAP1 diagnostic performance in MM diagnosis. We observed a fair diagnostic accuracy of BAP1 in diagnosing MM. The specificity of BAP1 detection was much higher than its sensitivity, and a good specificity-associated AUC was produced. However, the pooled estimates in present study must be interpreted with caution attributed to high heterogeneity and bias.

MM is a highly lethal neoplasm that is resistant to conventional treatments [30], while special treatments are not generally required in the most benign mesothelial processes, but follow-ups and patient reassurance are necessary. In the meantime, many MM patients worldwide are often misdiagnosed and cannot receive proper treatment. Therefore, accurate diagnosis of mesothelioma at an early stage is a critical clinical problem. However, distinguishing MM from non-MM can be very difficult, especially with an adenocarcinoma invading the pleural cavity. MM diagnosis relies on pathology, and a definitive diagnosis often requires IHC and histochemical mucin staining [31]. BAP1 plays a role in cycle-cell progression, DNA damage repair, gene expression regulation, and chromatin remodeling. Loss of BAP1 is responsible for the process of malignant mesothelioma, and the homozygous deletion of *BAP1* are associated with loss of IHC staining [32], thus leading to the high specificity of BAP1 in IHC. Increasing studies have been reported for evaluating BAP1 diagnostic utility in MM. A systematic meta-analysis by Walts [26] only estimated the weighted average percentage of correct results in effusion cytology specimens and did not offer a comprehensive analysis of BAP1 in diagnosing MM, and new, high-quality published data were also not included.

The results from our meta-analysis showed that the utility of BAP1 as a marker in the diagnosis of MM harbored a pooled sensitivity of 0.56 at a specificity of 1.00, meaning that loss of BAP1 immunostaining almost could rule in MM, but ruling BAP1 out as a marker seems to be premature. Low BAP1 sensitivity manifested lower performance for excluding MM, and patients with positive-staining for BAP1 remain at high risk for MM. The specificity, however, was strong and could have diagnostic potential for MM in spite of the relatively low sensitivity. Of note, cells expressing one wild-type copy of BAP1 retained IHC staining, and detection of BAP1 loss may be feasible for homozygous deletion of the BAP1. However, somatic *BAP1* mutations have also been found and initialized in the neoplasm of sporadic MM, which does not always cause the loss of IHC staining. The BAP1 antibody (C-4, Santa Cruz) is predicted to detect wild-type BAP1 and mutant forms, and the mutant forms might not be differentiated from wild-type, thus affecting the true positive in diagnosing MM. DOR, as an independent indicator, combines sensitivity and specificity data into one measure of performance [33] with higher values indicating better discriminatory test performance. In this study, meta-analysis displayed a DOR value of 1247.78 for BAP1 detection, revealing that BAP1 could effectively help discriminate MM from non-MM. Moreover, the SROC curve presents a comprehensive summary of diagnostic test performance, and the value of AUC for BAP1 was 0.72, thus showing a moderate diagnostic accuracy for MM.

For the DOR and SROC curve, applications in clinical practice are relatively difficult, but pooling likelihood ratios could provide more clinically meaningful measurements [34]. The value of PLR was 548.82, indicating that patients with MM harbored more than 548.82 times the possibility of BAP1 loss as the ones without MM. On the other hand, the NLR value of 0.44 suggested that patients with positive BAP1 staining still have a 44% chance of having MM; this percent is not low enough to eliminate MM. Taken together, BAP1 detection by IHC could be recommended as a valid diagnostic biomarker for MM, but it is not perfect. BAP1 needs to be combined with other markers (such as P16) in order to increase diagnostic accuracy in diagnosing MM, especially with regard to sensitivity.

Whether cytology is sufficient for a diagnosis of MM remains controversial. For this reason, we further performed a subgroup meta-analysis on the diagnostic performance of BAP1 resulting from cytological or histological specimens. We noted that the sensitivity, specificity, and NLR were nearly identical between two different specimens, but histological specimens showed superior diagnostic performance in terms of PLR, DOR, and AUC (Table 4). Meanwhile, an AUC value of 0.69 for BAP1 in cytology showed a low diagnostic accuracy for MM. Although loss of BAP1 expression in cytology could be applied to support mesothelioma diagnosis, histological specimens still have the added advantage, which is associated with the better diagnostic performance of histological diagnostic criteria when compared with cytological one.

MM has three histologic types, including EMM, BMM, and SMM, of which EMM is the most prevalent. A study reported that loss of BAP1 is rare in SMM [35]. Our data indicated that the sensitivity and DOR of BAP1 in EMM was higher than in other types. Despite an AUC value of 0.86, a sensitivity value of 0.07 causes the utility of BAP1 for diagnosing SMM to be limited. In addition, the value of AUC in BMM was 0.58, indicating low BAP1 diagnostic accuracy. Therefore, BAP1 harbors a better diagnostic performance in diagnosing EMM when compared with the other two subtypes, and loss of BAP1 is more inclined to support an EMM diagnosis. Of note, the superior diagnostic accuracy on EMM is associated with the high frequency of EMM, which may affect the results and cause diagnostic bias.

There was higher heterogeneity in the outcomes. Meta-analyses are often accompanied with different degrees of heterogeneity, and investigations of causes for heterogeneity are important goals. The subgroup analysis data have shown that sample origins and histological types were associated with diagnostic accuracy, which may cause a high degree of heterogeneity. In addition, several studies included in present meta-analysis set the optimized cut-off values on the basis of the observations in the particular populations. Different cut-off value settings may lead to a threshold effect, thus contributing to heterogeneity. Another reason for heterogeneity may be the variance in measurement and test matrices, racial differences, and high risk of publication bias.

The strengths of our systematic review and meta-analysis were consisted of three main points: 1.) Current meta-analysis almost pooled all recently published data regarding the utility of BAP1 in diagnosing MM and provided solid evidence for the diagnostic effect of this biomarker; 2.) Subgroup analysis concluded that the detection of BAP1 in histological specimens had better diagnostic performance than cytological ones; and 3.) We also came to the conclusion that BAP1 showed superior diagnostic accuracy in EMM than BMM or SMM.

There were also potential limitations that should be taken into consideration for this analysis. First, exclusion of studies published in the form of abstracts or letters to the editor (journal) may cause publication bias. In fact, we observed obvious publication bias in current study that may distort the conclusions. This bias might derive from the fact that studies reporting a significant effect tends to be more frequently accepted for publication, while this is the reverse in studies with negative conclusions. Second, the number of included studies was small, especially in subgroup. Moreover, subjects in some studies were small-scale, which decreased the power of the studies and may overestimate the true diagnostic accuracy. Third, random-effects modeling was used because of the significant heterogeneity in the analyses, which might have affected the results of the present study.

Despite these limitations, this meta-analysis suggested BAP1 IHC as a promising marker with extremely high specificity, and good DOR and area under curve in diagnosing malignant mesothelioma. However, BAP1 requires a combination with other biomarkers in order to improve the sensitivity.

## MATERIALS AND METHODS

### Search strategy

A comprehensive search was conducted including PubMed, Embase and the Cochrane Library from suitable studies to April 2017. The following keywords: (“*BRCA1–associated protein 1*” or “BAP1”) and (“mesothelin” or “mesothelioma” or “malignant mesothelioma” or “malignant mesothelin”) were searched. In addition, studies were also identified by manually searching the reference of the included studies and published reviews. Studies published only in the form of abstract or letter to journal were not included for the limited data.

### Study selection

We included studies that had to provide both specificity and sensitivity of BAP1 in MM diagnosis. Meanwhile, eligible studies must meet the following criteria: 1.) original studies assessing the diagnostic power of BAP1 for MM; 2.) containing true-positive, false-positive, true-negative, and false-negative data; and 3.) detection of BAP1 must be performed by IHC. Two reviewers independently evaluated the study eligibility. Disagreements were resolved through team consensus.

### Data extraction and quality assessment

Data were extracted using a standard collection form. Information from each study including author name, year of publication, clinicopathological features, patient characteristics, specificity and sensitivity data, and methodological quality were extracted by two independent reviewers. If several studies reported the overlap of patients, we chose large-scale or the best quality study to avoid duplication. Discrepancies were resolved through team consensus.

QUADAS-2 tool [37, 38] was applied to assess the methodological quality of selected studies. QUADAS-2 consists of four domains including patient selection, index test, reference standard, and flow and timing. Two reviewers independently evaluated the risk of bias and concerns regarding applicability, which were rating as “low”, “high” or “unclear” for one or more key domains.

### Statistical analyses

The meta-analysis was performed with STATA 12.0 software (Stata Corporation; Texas, USA). The diagnostic accuracy for BAP1 loss, together with 95% CI, was estimated by pooling sensitivity, specificity, PLR, NLR, and DOR. The sensitivity and specificity for the single test threshold identified for each study were used to plot a SROC curve [29, 39], which indicates the overall accuracy of diagnosis by AUC. AUC values of 0.5∼0.7, 0.7∼0.9 and 0.9∼ 1.0 represented low, moderate and high diagnostic accuracy, respectively. The random-effects modeling was used for meta-analysis [40].

*Q* test and *I*^2^ statistic were conducted to appraise heterogeneity, and *P* < 0.10 was considered significant heterogeneity. *I*^2^ values of 50%∼75% and 75%∼100% were considered to have moderate and high heterogeneity, respectively. Stata 12.0 software was used to make funnel plot and Egger test was conducted for evaluating publication bias where *P*-value < 0.05 suggested the publication bias existed. Funnel plots and Egger test were used to assess publication bias [41].

## Abbreviations

BAP1: BRCA1–associated protein 1
MM: malignant mesothelioma
IHC: imunohistochemistry
AUC: area under curve
DOR: diagnostic OR
NLR: negative likelihood ratio
PLR: positive likelihood ratio
MM: malignant mesothelioma
EMM: epithelioid malignant mesothelioma
BMM: biphasic malignant mesothelioma
SMM: sarcomatoid malignant mesothelioma
QUADAS-2: quality assessment of diagnostic accuracy studies-2
SROC: summary receiver operating characteristic.
